# Archaeal and Bacterial Diversity and Distribution Patterns in Mediterranean-Climate Vernal Pools of Mexico and the Western USA

**DOI:** 10.1007/s00248-021-01941-2

**Published:** 2021-12-31

**Authors:** Jorge A. Mandussí Montiel-Molina, Jason P. Sexton, A. Carolin Frank, J. Michael Beman

**Affiliations:** 1 Environmental Systems, Department of Life and Environmental Science, University of California Merced, North Lake Road 5200, Merced, CA 95343 USA; 2 Nativos de Las Californias A.C, Cuarto Balcón 15901, Balcón Las Huertas, Tijuana, Baja California 22116 México; 3 Jardín Botánico de San Quintín A.C, Gral. Esteban Cantú 200, Nuevo Baja California, San Quintín-Lazaro Cárdenas, Baja California 22930 México

**Keywords:** Ephemeral wetlands, Archaea, Bacteria, Inverse latitudinal gradient, Distance-decay, Environmental filtering

## Abstract

Biogeographic patterns in microorganisms are poorly understood, despite the importance of microbial communities for a range of ecosystem processes. Our knowledge of microbial ecology and biogeography is particularly deficient in rare and threatened ecosystems. We tested for three ecological patterns in microbial community composition within ephemeral wetlands—vernal pools—located across Baja California (Mexico) and California (USA): (1) habitat filtering; (2) a latitudinal diversity gradient; and (3) distance decay in community composition. Paired water and soil samples were collected along a latitudinal transect of vernal pools, and bacterial and archaeal communities were characterized using 16S rDNA sequencing. We identified two main microbial communities, with one community present in the soil matrix that included archaeal and bacterial soil taxa, and another community present in the overlying water that was dominated by common freshwater bacterial taxa. Aquatic microbial communities were more diverse in the north, and displayed a significant but inverted latitudinal diversity pattern. Aquatic communities also exhibited a significant distance-decay pattern, with geographic proximity, and precipitation explaining part of the community variation. Collectively these results indicate greater sensitivity to spatial and environmental variation in vernal pool aquatic microbial communities than in soil microbial communities. We conclude that vernal pool aquatic microbial communities can display distribution patterns similar to those exhibited by larger organisms, but differ in some key aspects, such as the latitudinal gradient in diversity.

**Supplementary Information:**

The online version contains supplementary material available at 10.1007/s00248-021-01941-2.

## Introduction

Microorganisms play multiple ecological roles in terrestrial and aquatic ecosystems, from acting as symbionts and parasites to regulating biogeochemical cycles. Quantifying how microbial communities assemble and how microbial taxa are distributed across ecosystems is therefore important to our understanding of ecological equilibrium. As such, a central question in microbial ecology is the degree to which microbes follow ecological patterns displayed by “macroorganisms” [9, 16, 17, 26, 35, 50]. Traditionally, microorganisms were thought to be widely distributed in all ecosystems due to their small size and high dispersal rates, with the environment responsible for “selecting” microorganisms according to their metabolic attributes [1, 44]. Niche theory predicts community composition as a result of environmental filtering, and irrespective of geographic proximity [42]—for example, salinity, temperature variation, oxygen limitation, and other particular conditions can strongly influence microbial community assembly in natural habitats [22, 39, 45, 49]. However, it is equally clear that a combination of historical events and contemporary factors are important in determining diversity and community composition, and that microbial communities can display broad biogeographic patterns [12, 26, 50]. Ultimately, microbial communities distributed within and across different ecosystems may be shaped by different assembly processes to differing degrees, providing insight into microbial community assembly in comparison to larger organisms.

Vernal pools are ideal systems in which to examine microbial community assembly given the inherent attributes of these ecosystems. Reflecting the climatic extremes typical of Mediterranean-climate ecosystems (moist and cold winters followed by hot and dry summers), vernal pools can form where water collects in shallow depressions with a high clay content and a deeper cemented layer (which prevents water percolation to the subsoil). Defined as temporary wetlands, and characterized by consecutive aquatic and terrestrial phases, vernal pools display seasonal transitions from completely flooded to totally desiccated soils [48]. During winters with sufficient precipitation, vernal pools fill with rainwater, creating water “islands” embedded in a surrounding terrestrial ecosystem. Vernal pools then progressively retract and eventually dry out in warmer months as evapotranspiration exceeds precipitation. Vernal pools therefore encompass both aquatic as well as soil habitats—which both vary strongly over the year, and may interact in various ways (e.g., via species that transition between the different environments, or via fluxes of carbon and nutrients). How microorganisms colonize these ecosystems, and tolerate the variations within them, directly addresses the roles of geography versus environment in affecting community assembly.

In soil, seasonal fluctuations from inundation to aridity likely create strong environmental variations. Under one extreme, saturated soils often reach anoxic conditions that may affect the distribution of microorganisms [7]. On the other extreme, dry and hot conditions during Mediterranean-type summers may require adaptations—for example, desiccation-adapted microalgae may persist dormant on dry stream beds until water flow resumes [43]. During the rainy season, inundated vernal pools resemble permanent aquatic ecosystems, however, the strong seasonality of Mediterranean-climate vernal pools shares attributes with other ephemeral aquatic ecosystems like seasonally flooded deltas, wetlands (pitlands), or floodplains [27, 37]. Freshwater ecosystems are generally inhabited by similar microbial groups (i.e., “typical freshwater bacteria”; Zwart et al. [51]) regardless of geographic location and characteristics, but with significant spatial and temporal variation within these groups [33]. These groups include typical freshwater Actinobacteria; alpha-, beta-, and gamma-proteobacteria; Cytophaga-Flavobateria-Bacteroides; and Verrucomicrobia [33, 51]. Whether similar freshwater bacterial groups colonize filled vernal pools—or whether they are inhabited by more specialized groups—is unknown. In either case, these communities may be used to examine biogeographic patterns in microorganisms. In particular, vernal pools can be considered habitable islands for microbes, and microbes may show geographical patterns in diversity and community composition [2, 14, 19, 32, 40].

In classical ecology, the latitudinal paradigm states that diversity gradually increases from the poles to the equator following a latitudinal gradient [23], and microbial ecologists have begun to examine whether microbes follow this pattern of biodiversity. However, whether such gradients result from a longer, more stable period of diversification in the tropics, higher speciation rates in the tropics, or lower extinction rates in comparison with temperate regions, still remains unresolved for larger organisms [29]. The presence and causes behind latitudinal patterns for microbes are even less clear. For example, Fuhrman et al. [15] detected a significant decrease in planktonic marine bacterial richness from the equator to the poles in the open ocean, but with notable variability in diversity at lower latitudes. Following a transect from mid-latitudes to the polar circles within lakes of South America and Antarctica, Schiaffino et al. [40] detected a decrease in evenness of microbial eukaryotes with decreasing latitude, but variations in richness were less clear. On land, Newsham et al. [32] detected a significant decrease in the richness of soil microfungi with increasing latitude along a 1650 km transect in Antarctica. These studies indicate that both aquatic and soil microbial communities may follow latitude-diversity gradients, but with notable differences in the shape and strength of these patterns—suggesting that additional research is needed. The distribution of vernal pools across the California province extends from 30 to 42°N (~ 2400 km), providing an opportunity to examine spatial patterns in diversity and community composition. Although this latitudinal range does not extend from the tropics to the poles, it provides a wide range of climatic conditions over which to test for the presence of diversity gradients.

Ecological studies have also observed a “distance-decay” pattern in many natural communities, in which community similarity decreases with increasing distance between locations [14]. Distance-decay patterns can be explained by the neutral theory that predicts community composition as a result of geographic proximity, where community similarity is driven by spatially limited dispersal, independent from environmental differences between sites [20, 31], on the other hand, environmental conditions play a key role by “filtering” some taxa, in that microbial communities that are closer together in space may experience similar environments. The degree to which distance-decay relationships are shaped by dispersal capabilities (spatial distance), environmental differences, or a combination of both, may differ among ecosystems. For example, community dissimilarity among microbial communities in alpine lakes can be partly explained by the distance between sites [19], whereas temporary cave-pool microbial communities are explained by the sunlight exposure and environmental properties of the water [41]. For microbial communities in vernal pools of the California province, there is a lack of studies focusing on these ecological features.

Under the premise that microorganisms can show distribution patterns similar to those exhibited by larger organisms, we tested the following hypotheses corresponding to specific paradigms established in classical and contemporary microbial ecology: (i) considering that vernal pools are subject to strong environmental selection [47], we hypothesized that water saturation and desiccation events will drive shifts in community composition from flooded pools to dry pools. Niche theory [42] states that the environment is the strongest driver for species to persist or recruit; in this context, moist and drought conditions may play a role in structuring microbial communities. If this is true, we expect to observe different microbial communities in saturated versus unsaturated soils, according to the seasonal-environmental variation in vernal pools, with a third community type present in the overlying water column. (ii) Considering that vernal pools are distributed latitudinally, we hypothesized the existence of a gradual increase in diversity of microorganisms along a transect from southern sites in Mexico to northern sites in the USA. (iii) In parallel, considering that vernal pools act as scattered or clustered habitat “islands” [47], we hypothesized that microbial communities will present spatial distribution patterns reflected in the community composition from each vernal pool. Thus, a distance-decay pattern is expected, where isolated vernal pools will have less similar microbial communities as a function of the space between pools. (iv) Finally, local environmental selection is likely to drive community composition as a result of the specific environmental properties across localities. We hypothesized that environmental variation in temperature and precipitation may be significant drivers of community similarity, explaining diversity between pools, sites, and regions, regardless of spatial proximity. Alternatively, dispersal capacity of microorganisms may be important in community assembly. If this is true, we expect to observe a relationship based mostly on spatial distances.

## Methods

### Study Area

**Fig. 1:**
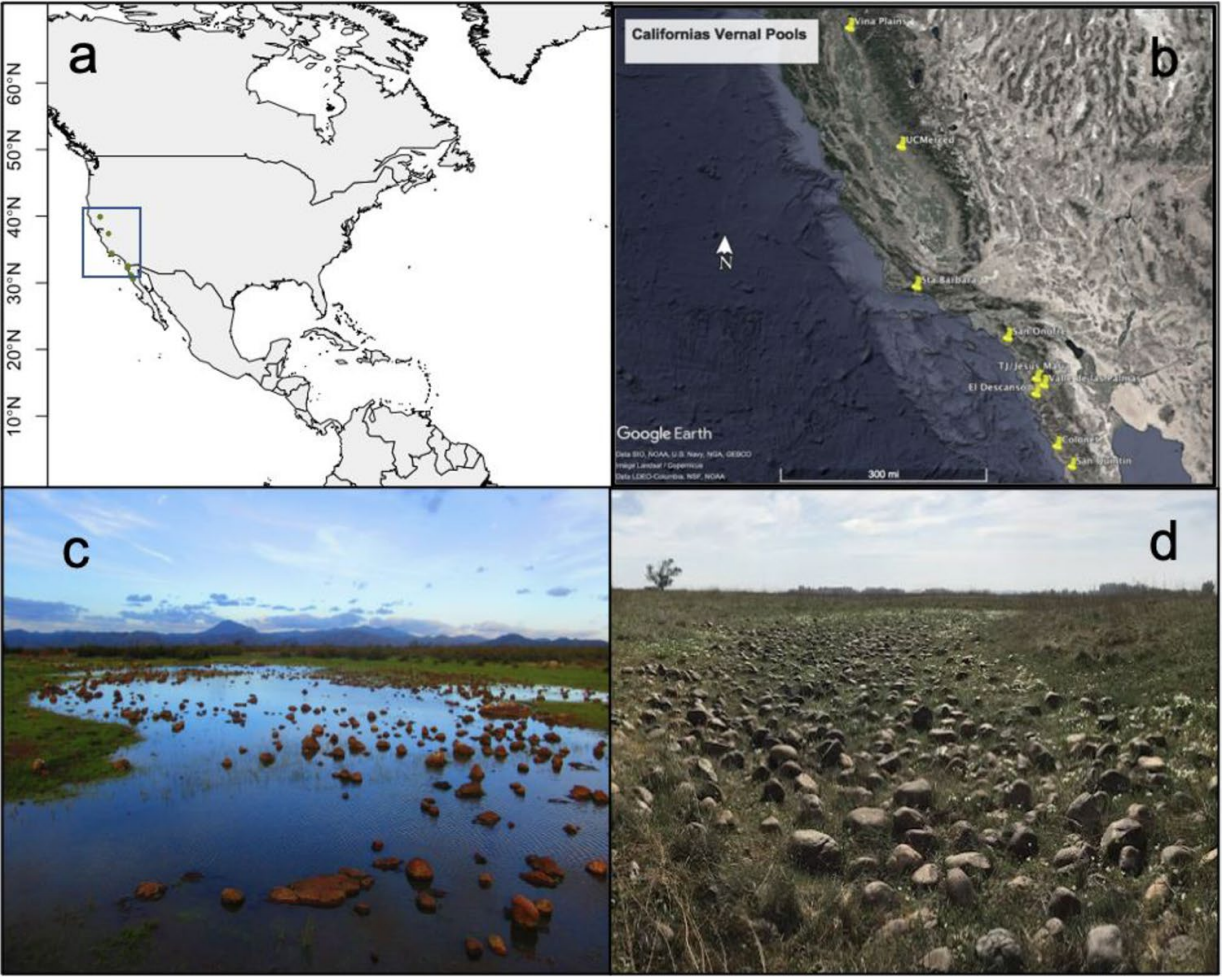
**a** North America Mediterranean-climate vernal pools. **b** Vernal pools extend along the Pacific West Coast in valleys and coastal mesas in California and Baja California. Climatic and geological features result in the formation of the vernal pools, which present **c** an aquatic phase characterized by flooding and soil saturation and **d** a terrestrial phase characterized by water evaporation and subsequent desiccation

As a Mediterranean-climate region, the study area is characterized by winter precipitation events and dry and hot summers. We sampled a latitudinal transect of ~ 1300 km, covering a significant portion of the geographical extension of the North American Mediterranean climate regime and the distribution of its vernal pools (Fig. 1). Sampling occurred in Baja California, Mexico, and California, USA. Each location varied in temperature and precipitation: temperature differences within the region range between 16° and 18**°** Celsius, and precipitation varies depending on subregions, but overall precipitation increases with latitude (data from BIOCLIM, Table 1). The vernal pools studied here belong to an “archipelago complex” (i.e., clusters of pools) or solitary vernal pools, often located at flat ground on top of coastal mesas or valleys. From north to south, locations were annotated alongside with their climate and geographical position: in California, USA, (1) Vina Plains, (2) Merced, (3) Santa Barbara; in Baja California, Mexico, (4) Mesa de Jesus Maria, Tijuana (5) El Descanso, (6) Valle de las Palmas, (7) Colonet (San Antonio del Mar), (8) Medina (Colonet mesa), and (9) Cerro las Torres, San Quintin.

**Table 1:** 30-year annual average temperature and precipitation of vernal pool regions and ID of the vernal pools sampled. Temperature (temp) is in Celsius and precipitation (precip) is in mm. Data were obtained from BIOCLIM

Vernal pool sites	Sample type collected	Sample ID number	Temp	Precip	Longitude	Latitude
Vina Plains i	Water	24	16.2	719	− 121.982942	39.901494
Vina Plains ii	Water	25	16.2	719	− 121.983788	39.899747
Merced i	Water	21	16.3	370	− 120.417208	37.376927
Merced ii	Water	22	16.3	370	− 120.417668	37.377302
Merced iii	Water	23	16.3	370	− 120.415969	37.375948
Santa Barbara i	Water	17	15.2	468	− 119.86534	34.415124
Santa Barbara ii	Water	18	15.2	468	− 119.866172	34.415841
Santa Barbara iii	Water	19	15.2	468	− 119.867498	34.415982
Santa Barbara iv	Water	20	15.2	468	− 119.868427	34.414142
Tijuana “Jesus Maria Mesa”	Water	11,49	16.9	277	− 116.833829	32.50431
	Wet soil	26				
	Dry soil	37				
El Descanso “Mesa”	Water	8,48	16.2	269	− 116.871812	32.177932
	Wet soil	27				
(✝)Valle De Las Palmas i	Dry soil	38	16.6	282	− 116.73994	32.39779
(✝)Valle De Las Palmas ii	Dry soil	39	16.6	282	− 116.74147	32.39682
Valle De Las Palmas iii	Water	2,14	16.3	340	− 116.64541	32.36915
	Wet soil	29				
Valle De Las Palmas iv	Water	3,15	16.3	340	− 116.64582	32.36913
	Wet soil	30				
	Dry soil	40				
Valle De Las Palmas v	Water	4,16	16.3	340	− 116.64631	32.36937
	Wet soil	31				
	Dry soil	41				
Colonet i	Water	9	16.6	242	− 116.28739	31.09416
	Wet soil	32				
	Dry soil	42				
Colonet ii	Water	12	16.6	242	− 116.28722	31.09355
	Wet soil	33				
	Dry soil	43				
Colonet iii	Water	13	16.6	242	− 116.28787	31.09249
	Wet soil	34				
	Dry soil	44				
Colonet iv	Dry soil	45	16.6	242	− 116.29275	31.05852
(✝)Colonet v	Dry soil	46	16.6	242	− 116.293954	31.027036
(✝)Colonet vi	Dry soil	47	16.6	242	− 116.293954	31.01739
Medina i	Water	5	16.6	242	− 116.28147	31.030867
Medina ii	Water	6	16.6	242	− 116.282222	31.030835
	Wet soil	35				
Medina iii	Water	7	16.6	242	− 116.283296	31.030569
	Wet soil	36				
San Quintin “Cerro las Torres”	Water	1,10	18.9	179	− 115.914153	30.584009
	Wet soil	28				

✝, vernal pools destroyed due to urbanization

### Sampling Design

To address our research interests regarding environmental selection and distribution patterns, we considered the first 15 cm from the soil surface to have a good representation of the soil microbiome [13]. We sampled each pool three times randomly from the center to the edge using a soil core, and homogenized the soil samples in sterile plastic bags. We collected one sample of 0.2 g of dry soil per pool in summer August 2016 for a total of 11 different pools. As a counterpart, we collected 0.2 g of “wet soil” from the same pools, but saturated with rainfall during the winter 2017, for a total of 11 pools. Unfortunately, four vernal pools were lost due to urbanization during our sampling period between summer and winter, and due to federal regulations, vernal pool soils were not possible to access in the USA, leaving three locations without soil and wet soil samples (Table 1). Both sample types were stored in 2 ml centrifuge tubes with dry ice before processing in the laboratory. In parallel, we also collected 100 ml of water per vernal pool during the winters of 2016 and 2017, for a total of 27 water samples analyzed in this study; some pools in Valle de las Palmas and Colonet regions were sampled twice during 2017 corresponding to different rain events. We filtered the microorganisms from the water samples using Waltham brand® filters of 0.23 μm pore size. After filtration, the filters were preserved in lysing tubes with a sucrose EDTA lysis solution and transported to the lab for DNA extractions [19]. Equipment was sterilized in between samples with 5% bleach, 100% ethanol, and rinsed with MilliQ water five times. Soil and filters were processed for DNA extractions with QIAGEN DNeasy^Ⓡ^ extraction kits. Final DNA aliquots with 50 μl per vernal pool were diluted to a final concentration of 20 ng per μl for further amplification and sequencing.

### Microbial Community Analysis

We examined dry soil, wet soil, and water samples via 16S rDNA amplicon sequencing targeting the regions V4–V5 using the universal primers 515F-Y and 926R, which have been shown to effectively capture bacterial and archaeal diversity [36, 38]. Sample barcoding, PCR amplification, and sequencing (Illumina MiSeq) were performed at Argonne National Laboratories in Chicago, IL, following Earth Microbiome Project protocols. Sequences were obtained on the Illumina MiSeqTM platform in a 2 × 250 bp paired-end run. Demultiplexing and assignment of the amplicon sequence variants (ASVs) were performed using the Qiime2 (v.2021.4) platform, and DADA 2 was used as a quality control for chimeric reads [5]. Samples were rarefied to a sampling depth of 4000 sequences for the diversity analyses since the majority of samples contained between 1–6 × 10^4^ sequence reads, with the exception of Vina Plains (ii) with 748 sequence reads; this water sample was omitted from the analysis. Alpha and beta diversity analyses were performed using dry soil, wet soil, and water samples; however, due to a lack of significant variation in the beta diversity of soil samples (see below), analyses to address distribution patterns were performed only with water samples.

### Diversity Analysis

Analyses of amplicon sequence variants (ASVs) were performed using the platform Qiime2 (v.2021.4) designed for microbiome bioinformatics analyses; R studio (v.1.3.1093) packages qiime2-v0.99.1, phyloseq-v1.34.00, ggplot-v3.3.3, microbiome-v 1.14.0 for additional statistical analyses and figures; and PASTv4.0 was used in parallel for ASV’s diversity analyses [3, 4, 18, 25, 28, 46]. Alpha diversity metrics included Richness (observed taxa), Shannon (richness/evenness), Simpson (richness/abundances), Pielou (evenness), and ACE (richness and sampling coverage), and all of them were performed with Qiime2 and phyloseq. The Kruskall-Wallis test was used to analyze differences in alpha diversity metrics between dry soil, wet soils, and water samples. Beta diversity was analyzed in Qiime2 and phyloseq using Bray–Curtis and unweighted Unifrac metrics of community dissimilarity; environmental information was included in community analyses using PCA and NMDS ordination methods. Permutation analysis of variance (PERMANOVA) and analysis of similarity (ANOSIM) (999 permutations) were used to correlate dissimilarity matrices for grouping significance and to make pairwise comparisons between sample types (dry soil vs. water, wet soil vs. water, and dry vs. wet soil). Alpha diversity was correlated with latitude and beta diversity values between water samples were correlated (r) against spatial distances between sites, with *P* < 0.05 used to determine significance. Mantel tests (rho) were performed to correlate rarified taxa abundance similarity matrix values with temperature, precipitation, and geographical distance matrices.

## Results

We tested multiple hypotheses through analysis of microbial communities inhabiting vernal pools. Overall, we found that the soil matrix and water column represented two adjacent but distinct habitat types, with different communities occupying different niches (rejecting the possibility of a single community transitory between the soil matrix and water column). Aquatic microbial communities displayed several significant patterns. We found a gradual increase in the diversity of microorganisms from southern sites in Mexico to northern sites in the USA, as well as a significant distance-decay pattern—with spatial proximity and environmental differences at site level explaining community similarity across vernal pools. Below we report observed patterns in microbial diversity and composition within vernal pools, followed by analysis of these patterns.

### Variations in Microbial Community Composition Between Soils, Wet soils, and Water in Vernal Pools

**Fig. 2:**
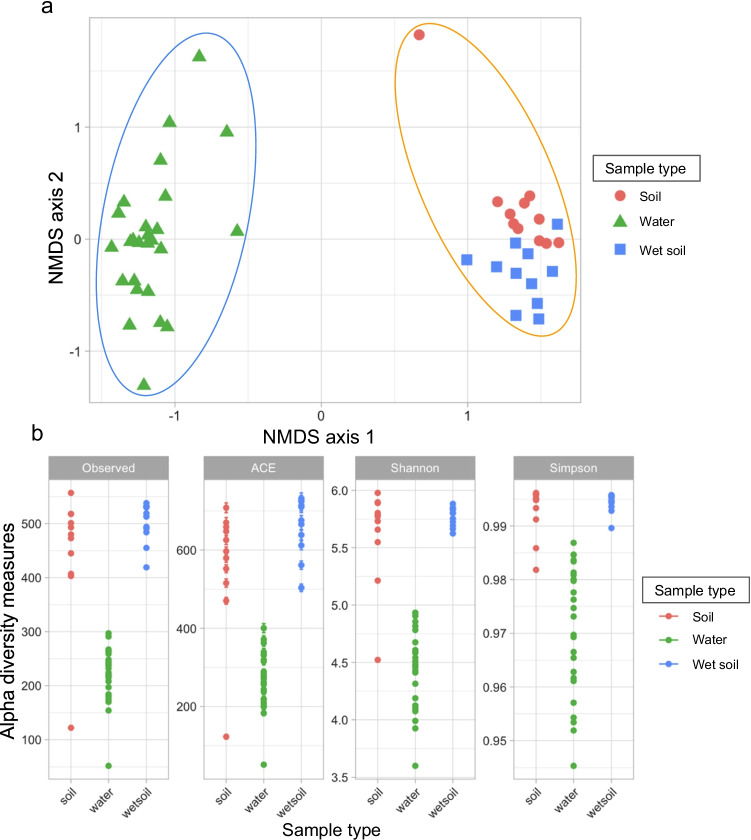
Microbial community analysis. **a** Beta Diversity: NMDS ordination analysis based on Bray–Curtis similarity coefficients. Soils and water have distinct microbial communities. **b** Alpha diversity by four different measures and by sample type. Soils (dry and wet) showed higher diversity in comparison with the water column: soil–water *H* = 18.9, *P* = 0.000013; wet soil–water *H* = 22.5, *P* = 0.000002; soil-wet soil *H* = 0.13, *P* = 0.71

Analysis of microbial communities within vernal pools spread throughout California, USA, and northern Baja California, Mexico, revealed multiple patterns in composition and diversity (Fig. 2). First, soil and water samples showed significant differences in microbial communities (Table 2). Beta diversity analysis (NMDS based on Bray–Curtis) also showed greater variability across aquatic microbial communities in comparison with soil microbial communities (Fig. 2a). Soil communities were more similar than water samples (outside of a few exceptions; see below), but with additional differences between dry and wet soils detected by PERMANOVA and ANOSIM (Table 2). Alpha diversity analyses of 22 soil samples, including dry soils and wet soils, were similar for all metrics of richness and abundance—suggesting that dry and wet soils host similarly diverse microbial communities across different sites (Fig. 2b). In contrast, aquatic communities displayed lower alpha diversity than soils (Fig. 2b), but greater beta diversity between these communities (Fig. 2a).

**Table 2:** Group significance by permutation analysis of variance (PERMANOVA) and analysis of similarity (ANOSIM) between sample types

	Dry soil–water	Wet soil–soil	Water–wet soil
**PERMANOVA**			
Bray–Curtis	Pseudo *F* = 5.265952, *P* = 0.001	Pseudo *F* = 1.633980, *P* = 0.002	Pseudo *F* = 4.753768, *P* = 0.001
Unweighted-Unifrac	Pseudo *F* = 7.857370, *P* = 0.001	Pseudo *F* = 1.632099, *P* = 0.001	Pseudo *F* = 8.048377, *P* = 0.001
**ANOSIM**			
Bray–Curtis	*R* = 0.98, *P* = 0.001	*R* = 0.21, *P* = 0.002	*R* = 0.97, *P* = 0.001
Unweighted-Unifrac	*R* = 0.89, *P* = 0.001	*R* = 0.28, *P* = 0.001	*R* = 0.90, *P* = 0.001

### Microbial Taxa in Soil and Water

**Fig. 3:**
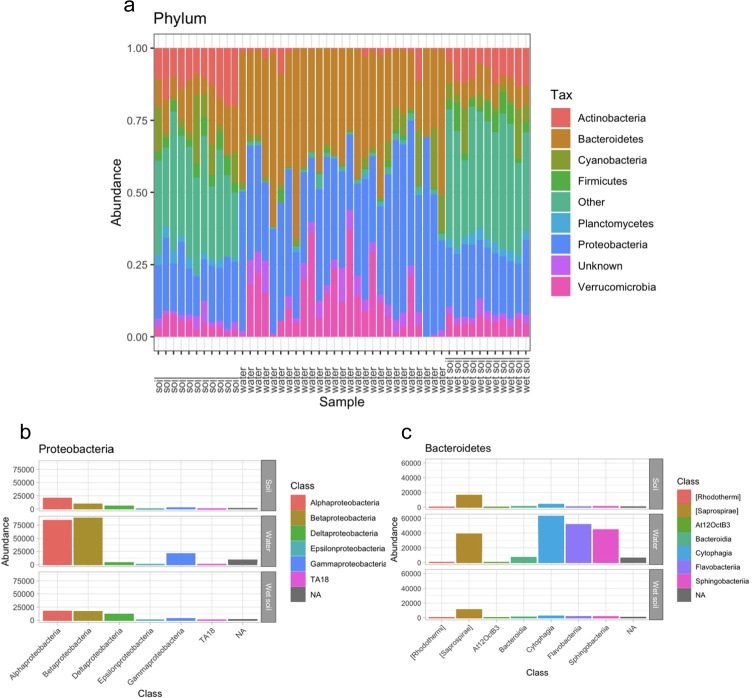
Taxonomic composition in water and soil samples, illustrating greater differences between water and soils than between wet and dry soils. **a** The relative abundance at phylum level across sample types; **b** Proteobacteria abundances across sample types; and **c** Bacteroidetes abundances across sample types

Bacteria were present in both soils and water, but with marked differences between sample types (as the beta diversity analyses above indicate). We found 42 phyla in total, and the most abundant were Proteobacteria and Bacteroidetes (Fig. 3a). Water was dominated by a few phyla, primarily Bacteroidetes and Proteobacteria, followed by Verrucomicrobia and Actinobacteria in lower proportions.

Soil was dominated by Proteobacteria, followed by Actinobacteria, Acidobacteria, Bacteroidetes, Cyanobacteria, Firmicutes, Verrucomicrobia, Planctomycetes, and Gemmatimonadetes. Our analysis revealed Fusobacteria as a rare phylum associated only with water samples; rare phyla associated with soil samples included AD3, BHI80139, GOUTA4, Kazan 3B28, MVP21, OP3WS2, WS3, WS4, WS6, and WWE1. Differences between soil and water were more clearly evident at the class level, with alpha-, beta-, and gamma-proteobacteria being more common in water than in either dry or wet soil (Fig. 3b). Within the Bacteroidetes phylum, Cytophagia, Flavobacteria, and Sphingobacteria were likewise more common in water (Fig. 3c). In contrast, soils showed a more even distribution of all classes of Proteobacteria and Bacteroidetes.

**Fig. 4:**
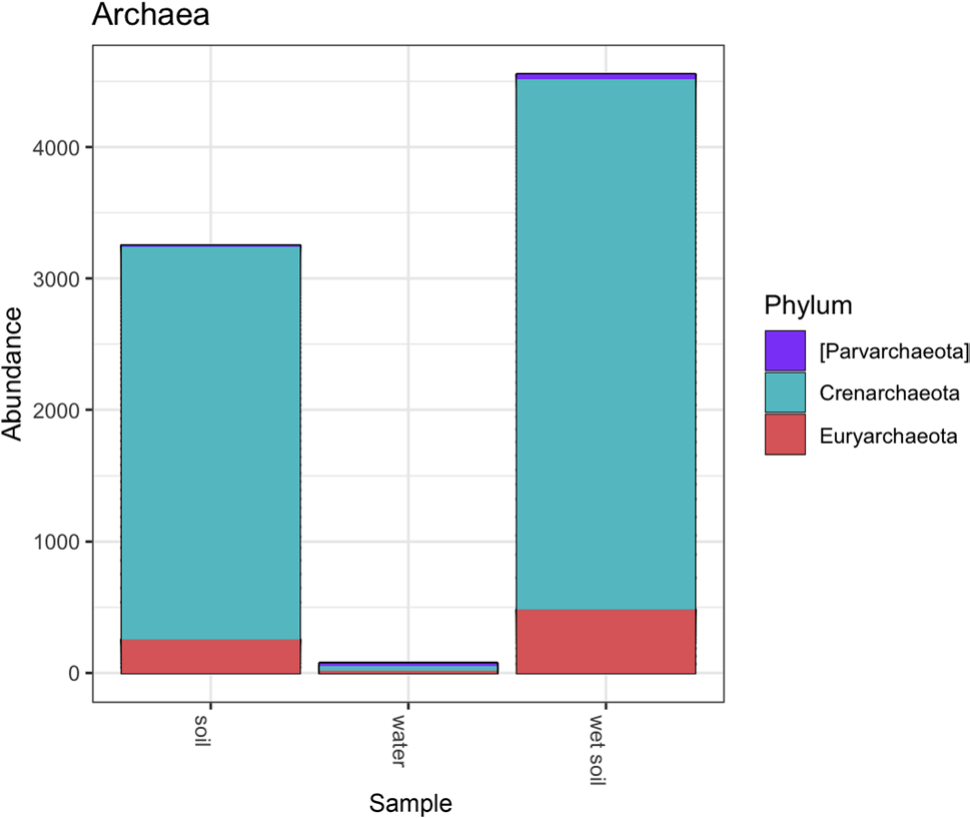
Taxa abundance of Archaea at phylum level between soil, wet soil, and water samples. The brackets indicate the taxonomic name is contested, morphology-based taxonomy doesn’t always align with phylogeny

Archaea were detected almost exclusively in soils, comprising the phyla Crenarchaeota, Euryarchaeota, and Pavarcheota. Archaea being mostly limited to soils suggests that aquatic archaea are less likely to inhabit vernal pool waters (Fig. 4). At the class level, archaeal diversity was dominated by Thaumarchaeota followed by Methanomicrobia, Thermoplasmata, MCG, and Parvarchaea. Overall, diversity was higher in soils than water, and dominant groups appeared consistently, even within those samples with lower numbers of sequence reads.

### The Latitudinal Diversity Gradient in Vernal Pool Microbial Communities

**Fig. 5:**
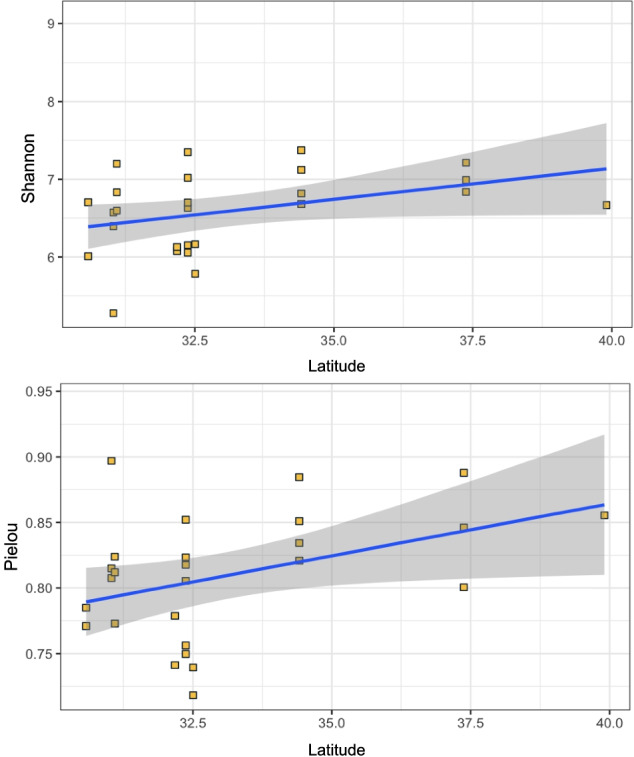
Diversity along a latitudinal gradient is inverted for aquatic prokaryotes in vernal pools. The scatter plots show Shannon index (**a**) and Pielou’s evenness index (**b**) in vernal pools plotted across latitude (yellow dots)

We tested for several biogeographic patterns in vernal pool microbial communities. First, the general increase in biodiversity from the poles to the equator is what defines the concept of the latitudinal diversity gradient, and community composition is expected to transition gradually in sites along this gradient. We found a significant latitudinal diversity gradient in vernal pool microbes; however, this was only significant for aquatic communities, and was also inverted, with higher diversity at higher latitudes (Fig. 4). Additionally, this pattern was only observed for diversity metrics based on evenness, with a significant relationship between latitude and Pielou’s evenness, and a nearly significant relationship for the Shannon index (Table 3, Fig. 5).

**Table 3:** Correlation coefficients between diversity and latitude; significant Pearson’s *r* for the alpha diversity-latitude relationship indicate a positive relationship between latitude and Pielou’s evenness

	**ASV richness**	**Shannon**	**Pielou**	**ACE**
Significance ***p***	0.95	**0.055**	**0.036**	0.785
Distribution ***t***	− 0.054941	**2.0131**	**2.2114**	− 0.27562
***Pearson’s r***	− 0.01121413	**0.38**	**0.411**	− 0.05617155

### Distance Decay: Dispersal Capabilities vs Environmental Selection

**Fig. 6:**
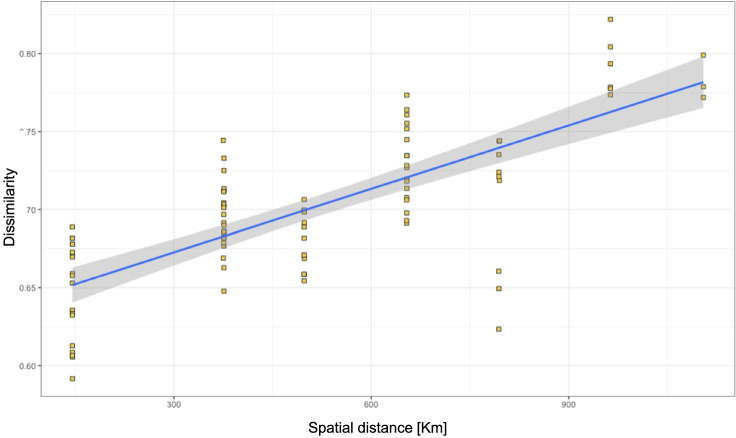
Dissimilarity in aquatic microbial communities in vernal pools increases with distance between sites; each point represents a specific value given by the Bray–Curtis dissimilarity index between two vernal pool microbial communities at varied distances

Our results also showed community composition strongly explained by the distances among sites (*R*^2^ = 0.5625; *P* < 0.01 Fig. 6), suggesting that community isolation is driven by dispersal limitation or environmental differences between sites. To test whether dispersal limitation (geographical distance) or environmental filtering were drivers of community composition, we analyzed the correlation (rho) between climatic parameters, spatial distances, and community dissimilarity indices. We tested if the microbial community composition was correlated with mean annual precipitation and temperature. Temperature did not influence community composition. The Mantel test based on Spearman’s rank correlation (permutations = 9999) between mean precipitation and taxa distribution (Bray–Curtis dissimilarity) showed significant correlation including all samples, soil and water (*r*: 0.1269, *P* = 0.0249). Precipitation also explained the microbial community composition for water samples (*r*: 0.4324, *P* = 8e-04). On the other hand, geographical distance (*r*: 0.32, *P* = 0 0.02)—which addresses the influence of dispersal limitation—also significantly explained the assembly of communities (Table 4). A combination of local precipitation and geographical distance between sites was therefore significantly related to community composition.

**Table 4:** Mantel test summary: correlation between similarity matrix based on rarified abundances of microbial taxa, and precipitation or geographical distances (spatial distances)

Matrix analyzed	(rho)	Significance *p*	Permutations
Precipitation/total.abund	*r*: 0.1269	*p* = 0.0249	9999
Precipitation/water.abund	*r*: 0.4324	*p* = 8e-04	9999
Geographical.distance/water.abund	*r*: 0.3203	*p* = 0.0203	9999

## Discussion

Our analysis of microbial communities in vernal pools provides additional evidence that microorganisms can display the same natural patterns as larger organisms [12, 26, 50]. However, some interesting attributes were not expected: for example, despite strong seasonal variations in vernal pools, their aquatic microbial communities were similar to other freshwater ecosystems. Vernal pools evidently represent isolated aquatic “islands” surrounded by completely terrestrial ecosystems. This raises interesting questions, such as where, when, and how freshwater microbes colonize vernal pools. Related to this, the geographical distribution of pools across Baja California and California was important for aquatic microbial community assembly, but not for soil microbial communities. Several biogeographical patterns were visible and significant within water, despite some limitations and caveats that we discuss below.

### Implications of Vernal Pool Microbial Communities Resembling Other Freshwater Ecosystems

We expected that vernal pool variability (desiccation to inundation) could potentially select for unique taxa, but we found that vernal pool microbial communities in water were dominated by “typical” freshwater bacterial taxa [21, 33, 34, 51]. These groups are commonly found across a wide range of freshwater ecosystems, and specifically include Bacteroidetes and Proteobacteria (Fig. 3). For example, we found that eight of the most common ASVs in water (each > 1% of all sequences, with higher abundances in some individual pools) were 98.3–100% identical to database sequences found in other freshwater ecosystems (Table S1). This included sequences from a large-scale study of freshwater bacterioplankton [8], as well as multiple whole genomes recovered from other freshwater ecosystems. Actinobacteria are also common in freshwater [33], and were present (albeit less abundant overall) in vernal pools. Even within those samples with lower sequence reads (water sample VP025), these common freshwater taxa were dominant.

These findings suggest that common freshwater microbes are able to colonize vernal pools each year in spite of the ephemeral nature of the pools. How exactly vernal pools are populated by common freshwater taxa, and whether vernal pools contain endemic archaeal and bacterial communities are interesting questions worthy of additional study and experimentation. Freshwater bacteria are notably widespread [51], and the attributes of vernal pools may help provide insight into their broad geographic distribution and the connectivity between freshwater ecosystems via animals or air. For example, possible transport pathways could involve larger organisms that use the vernal pools as a place of rest and breeding, such as migratory birds. Amphibians breeding in stock ponds might also play a role as a vector for microbial taxa, transporting and connecting permanent freshwaters with vernal pools. Additionally, rain might play an important role in transporting microorganisms [11]. On the other hand, these Mediterranean-climate vernal pools have a cemented layer preventing interchangeable flows from subsoil to surface, which reduces the possibility of groundwater connectivity. Considering the impact of anthropogenic activities over the landscape—where some vernal pools have been transformed into stock ponds, and other human-made water reservoirs are located nearby—anthropogenic activities may also be relevant in the context of microbial distributions and their alterations [22]. Finally, some taxa may also disperse widely by wind, and environmental constraints between habitats might shape microbial communities [10].

### Lack of Significant Biogeographic Patterns in Soil Microbial Communities

A main goal of this research was to determine if microbial communities in vernal pools show distribution patterns previously recorded in other microbial systems and for larger organisms. We detected several patterns in microbial communities (e.g., a significant latitude-diversity gradient and distance decay)—but these patterns were only significant for aquatic communities. In the case of soil, our results indicate that conditions in vernal pool soils select for consistent microbial communities across distant locations, with the exception of one sample located at the southern edge of our region of study (soil sample 46-ColonetV, Fig. 2 outlier). Several factors might have obscured our ability to detect significant patterns in soil, if present. One issue is the presence of relic DNA coming from extracellular or non-intact cells, which can account for 40 to 80% of prokaryotic DNA in soils, and can persist in soil for weeks to years [24]. Another possibility is that precipitation events have an impact on the microbial community by “resetting” a well-established microbial community. In combination with physicochemical properties (i.e., cation exchange capacity and pH), this could have a direct effect on the recovery of eDNA [6, 24]. Microbial adaptations to survive long periods of desiccation and the transition from flooding to desiccation could result in low variation from one phase to another [43]. Finally, our soil sampling was necessarily more spatially limited than our water sampling, and additional sampling could capture greater community variation. Including other molecular techniques such as metatranscriptomic sequencing may also help reveal additional patterns, especially within soil microbial communities.

### Inverted Latitudinal Gradient

One significant pattern detected in vernal pool water was a significant latitude-diversity relationship, but this was inverted, with higher evenness at higher latitudes. Some ecological explanations for such inverted gradients rely on specific interactions between taxa—for example, predation—and theories of energy around environmental systems [30]. We suggest that the most likely explanation for this relationship is related to precipitation, as much higher precipitation rates occur at higher latitudes along our sampling transect, previous studies have addressed a climate-latitudinal diversity gradient in other temporal freshwater bodies [23]. As a result, vernal pools in southern latitudes have shorter periods of inundation, and dispersal may be diminished due to the absence of nearby water bodies. This may result in more uneven communities. Based on the limited energy hypothesis behind latitudinal diversity—which states that sunlight is directly correlated with plant productivity and species biodiversity [30]—another possible explanation is that higher primary production in Northern California is a reflection of longer growth periods (due to greater precipitation rates), with inundated vernal pools lasting longer and favoring higher diversity. Moreover, the inverse prokaryotic diversity gradient observed among these study sites may be associated with eukaryotic diversity patterns, which we did not evaluate.

### Significant Distance Decay in Vernal Pool Aquatic Microbial Communities

Like islands in the ocean, vernal pools are ideal for understanding ecological and evolutionary processes that shape biodiversity. The pattern we described in this study has been found in freshwater bodies, where the existing pattern of dissimilarity (community isolation) is a product of both environmental differences and the spatial distance between sites [19, 41]. Our results also partially explain the distance-decay pattern based on both climatic variation and dispersal capabilities, indicating that environmental selection and microbial dispersal are both important for vernal pool aquatic microbial community assembly. Further studies should directly address if surrounding freshwater bodies, precipitation, atmospheric deposition, or large organisms provide vernal pools with freshwater taxa. Related to this, interspecific interactions—such as predation or other symbiotic relationships—may also affect vernal pool microbial communities to varying degrees. Stochastic events and anthropogenic influences may also be important [22, 50], for example, across our sampling gradients, pools at southern latitudes remain more pristine. Given their inherent attributes and significant distance decay in microbial community similarity, vernal pools appear to be an effective study system for studying microbial community assembly.

## Conclusions

This research is the first formal attempt to characterize and quantify microbial communities in Mediterranean-climate vernal pools in North America, providing initial insight into the microbial ecology of these endangered ecosystems. Overall, our study indicates that environmental selection plays an important role in defining distinct vernal pool microbial communities in soil and water. Aquatic communities in vernal pools exhibit a non-traditional latitudinal diversity pattern, which may be partially explained by precipitation patterns. Dispersal limitation is important as well, as a combination of spatial and environmental variation shaping the assembly of vernal pool aquatic microbial communities. Whether such patterns are consistent over longer time periods, and what mechanisms are involved to assemble community differences observed here are important future research avenues. Vernal pools are well-known for being inhabited by organisms adapted to both aquatic and terrestrial conditions (e.g., plants), with life cycles modified in order to survive shifts of inundation and total desiccation in a short period of time. Occasionally this transition occurs quickly, with intermittent precipitation and evapotranspiration happening rapidly within days. Exploring these transitions at different temporal scales may provide insights about “amphibious behavior” in microorganisms inhabiting these ecosystems—i.e., the ability to survive in both soil and water. Finally, additional spatial, temporal, and biological (e.g., plant and animal hosts and vectors) sampling may reveal new biological discoveries in these endangered vernal pool ecosystems.

## Supplementary Information

Below is the link to the electronic supplementary material.

Supplementary file1 (DOCX 7 KB)

## Data Availability

DRYAD.
