# Supplementary material for: Archaeal and Bacterial Diversity and Distribution Patterns in Mediterranean-Climate Vernal Pools of Mexico and the Western USA
Source: Microb Ecol. 2021 Dec 31;85(1):24–36. doi: 10.1007/s00248-021-01941-2 (PMC8718339; doi:10.1007/s00248-021-01941-2)
Supplement: Supplementary file 1 — Supplementary file1 (DOCX 7 KB) [file 248_2021_1941_MOESM1_ESM.docx]

**Table S1** Sequence identity of abundant aquatic ASVs to database sequences.

| ***ASV*** | ***Taxonomic affiliation*** | ***Genbank Accession*** | ***Identity (%)*** |
| --- | --- | --- | --- |
| 333c4bef102ebd8c41d9f3012937a8ef | Verrucomicrobia | MH112360 | 98.9 |
| d459c6a805c8512605fef8656ca0bf9a | Verrucomicrobia | FJ694301 | 98.3 |
| 9fcd6a2538aa25223fb828d5fffa26e9 | Sphingomonas | MZ976831 | 100 |
| c7de82d60809c2abae1ff7c61adedfec | Flavobacteria (genome) | AP025185 | 100 |
| 878cc93048466edf82856c4cf5e20517 | Beta-proteobacteria | FJ694257 | 100 |
| 4bba88bb794faeb8486f292406ea2641 | Gemmobacter (genome) | CP028918 | 100 |
| 1b24e60fca2c1ce5d0e34cab586c4537 | Polaromonas | MT067339 | 100 |
| 1ceb504a01db919823bd7d01b5699038 | Polynucelobacter (genome) | CP028942 | 100 |
